# Kcnj16 knockout produces audiogenic seizures in the Dahl salt-sensitive rat

**DOI:** 10.1172/jci.insight.143251

**Published:** 2021-01-11

**Authors:** Anna D. Manis, Oleg Palygin, Elena Isaeva, Vladislav Levchenko, Peter S. LaViolette, Tengis S. Pavlov, Matthew R. Hodges, Alexander Staruschenko

**Affiliations:** 1 Department of Physiology,; 2 Cardiovascular Center, and; 3 Department of Radiology, Medical College of Wisconsin, Milwaukee, Wisconsin, USA.; 4 Clement J. Zablocki VA Medical Center, Milwaukee, Wisconsin, USA.

**Keywords:** Neuroscience, Epilepsy, Ion channels, Potassium channels

## Abstract

K_ir_5.1 is an inwardly rectifying potassium (K_ir_) channel subunit abundantly expressed in the kidney and brain. We previously established the physiologic consequences of a *Kcnj16* (gene encoding K_ir_5.1) knockout in the Dahl salt-sensitive rat (SS^Kcnj16–/–^), which caused electrolyte/pH dysregulation and high-salt diet–induced mortality. Since K_ir_ channel gene mutations may alter neuronal excitability and are linked to human seizure disorders, we hypothesized that SS^Kcnj16–/–^ rats would exhibit neurological phenotypes, including increased susceptibility to seizures. SS^Kcnj16–/–^ rats exhibited increased light sensitivity (fMRI) and reproducible sound-induced tonic-clonic audiogenic seizures confirmed by electroencephalography. Repeated seizure induction altered behavior, exacerbated hypokalemia, and led to approximately 38% mortality in male SS^Kcnj16–/–^ rats. Dietary potassium supplementation did not prevent audiogenic seizures but mitigated hypokalemia and prevented mortality induced by repeated seizures. These results reveal a distinct, nonredundant role for K_ir_5.1 channels in the brain, introduce a rat model of audiogenic seizures, and suggest that yet-to-be identified mutations in *Kcnj16* may cause or contribute to seizure disorders.

## Graphical abstract

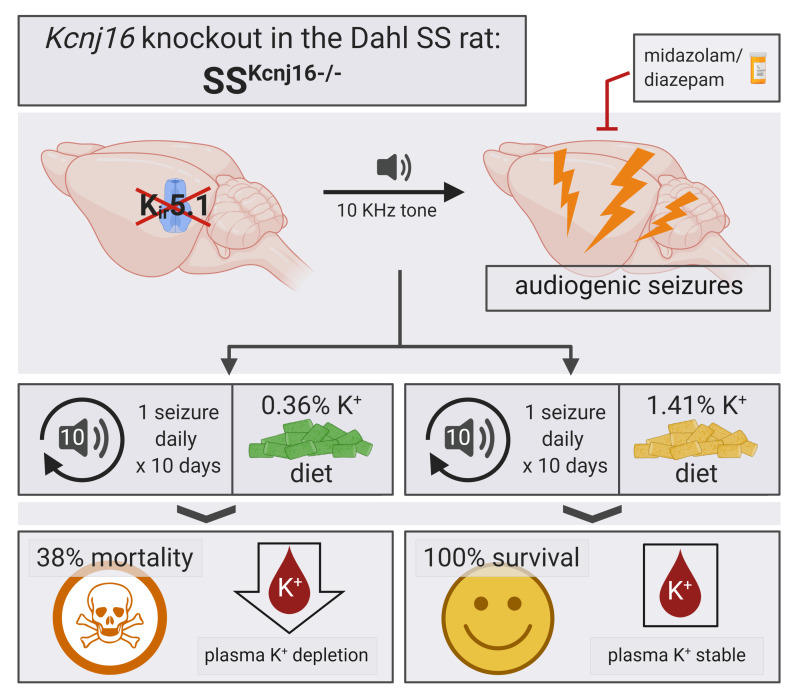

## Introduction

Epilepsy is among the most common neurological disorders and is characterized by a sustained increased susceptibility to hyperexcitable or hypersynchronized electrical activity in the brain, which can produce recurrent seizures (1, 2). Epilepsy affects all ages, genders, and nationalities; however, many patients with epilepsy are not receiving or are unable to access appropriate treatment. Of the patients receiving proper care, only two-thirds are responsive to treatment, leaving about 13 million patients refractory to the more than 30 pharmaceuticals available to treat seizures (3, 4). Epilepsy represents a wide array of seizure disorders with substantial phenotypic and genetic heterogeneity, which can obscure understanding of the mechanisms behind refractory epilepsy and create a barrier to the development of effective therapeutic strategies. Moreover, epilepsy is a disease of complex and multifactorial etiology, with most cases being considered idiopathic. Although only a few forms of seizure disorders are known to be monogenic in origin, the majority of idiopathic epilepsies are hypothesized to have a genetic basis (5, 6). Electrical activity in the brain is governed by the movement of ions across the cell membrane, which is mediated by ion channels. Seizure disorders are commonly associated with impairments in electrolyte homeostasis, and mutations in genes encoding ion channels are some of the few molecular causes that have been identified (7). Animal models are necessary to elucidate the physiological consequences of mutations in specific ion channel genes, determine the effect of these mutations on neurological function and potential contribution to epileptogenesis, and to ultimately identify and evaluate novel therapeutic targets for the treatment of epilepsy.

Precise regulation of potassium (K^+^) on both a cellular and systemic level is imperative for neurological function and is governed by several types of K^+^ channels expressed on the plasma membrane. Inwardly rectifying K^+^ (K_ir_) channels are evolutionarily ancient channels expressed in nearly all cell and tissue types, which contribute to diverse cellular and physiological functions, including maintenance of the resting membrane potential, modulation of cellular excitability, and regulation of whole-body electrolyte homeostasis (8). Functional K_ir_ channels comprise subunits expressed by 17 known *KCNJ* genes in either homotetrameric or heterotetrameric assemblies. Due to their ubiquitous expression and crucial function in cellular homeostasis, K_ir_ channelopathies affect multiple organ systems and can be severe or lethal. Mutations in K_ir_ channel genes have been associated with neurological dysfunction and seizure disorders, including a complex syndrome known as epilepsy, ataxia, sensorineural deafness, tubulopathy/seizures, sensorineural deafness, ataxia, mental retardation, and electrolyte imbalance (EAST/SeSAME), which results from mutations in *KCNJ10* (gene encoding K_ir_4.1) (9, 10). It has been postulated that causative missense mutations in *KCNJ10* prevent K_ir_4.1 from forming a heterotetramer with K_ir_5.1 (*KCNJ16*), and it is the loss of the more strongly rectifying heteromeric K_ir_4.1/K_ir_5.1 channel that produces the severe phenotype in this rare syndrome (11). However, the effect of the loss of function of *Kcnj16* on neurological dysfunction and epilepsy has not been evaluated. Similar to K_ir_4.1, K_ir_5.1 (encoded by *Kcnj16*) is a K_ir_ channel subunit highly expressed in the kidney and brain. However, unlike K_ir_4.1, K_ir_5.1 channel subunits are not thought to form a functional homomeric channel in vivo. It is clear that K_ir_4.1 and K_ir_5.1 proteins coassemble to form a functional, pH-sensitive, and heterotetrameric channel in the kidney, but this has not been unequivocally shown in the brain (12, 13). Although little is known about the physiological roles of K_ir_5.1, it has been shown to contribute to the regulation of intrinsic and extrinsic K^+^ homeostasis, acid-base equilibrium, and other homeostatic systems (14–17). Thus, the composition and function of K_ir_5.1-containing channels in the brain remain unclear, and the potential contributions of mutations in *KCNJ16* to neurological dysfunction and human disease are unknown.

To investigate the neurological, physiological, and pathophysiological role of K_ir_5.1, we used a recently generated and validated global knockout of *Kcnj16* on the Dahl salt-sensitive (SS) rat background (SS^Kcnj16–/–^ rats) (18). Our previous work indicated that the K_ir_5.1 mutation in the kidney led to a complex cardiorenal phenotype, including low body weight, renin-angiotensin-aldosterone system dysregulation, hypokalemia, and protection from salt-induced hypertension with dietary K^+^ supplementation (18, 19). SS^Kcnj16–/–^ rats also showed deficits in acute (CNS-respiratory) and chronic (renal) acid/base dysregulation (20). These findings suggest that K_ir_5.1 plays a nonredundant role in fundamental physiological homeostasis (18, 19, 21–23). Given that K_ir_5.1 is highly expressed in the CNS, loss of K_ir_5.1 expression may destabilize resting membrane potential, thereby altering neuronal excitability and resulting in additional neurological phenotypes. Herein, we investigated the neurological phenotype of the SS^Kcnj16–/–^ rat to build understanding of K_ir_5.1 function in the brain. The data support the hypothesis that K_ir_5.1 loss increases neuronal excitability, thereby increasing susceptibility to sound-induced seizures, which, when repeated, led to ictal apnea, hypokalemia, and high mortality rates, which can be prevented with dietary K^+^ supplementation. This model represents to our knowledge the first rat model of sound-induced seizures with a known genetic cause; this model will advance our understanding of seizure disorders and may elucidate the mechanisms behind the high risk of sudden unexpected death in epilepsy (SUDEP) in patients with refractory epilepsies.

## Results

### Mutation in Kcnj16 results in behavioral and electrographic audiogenic seizures.

**Figure 1:**
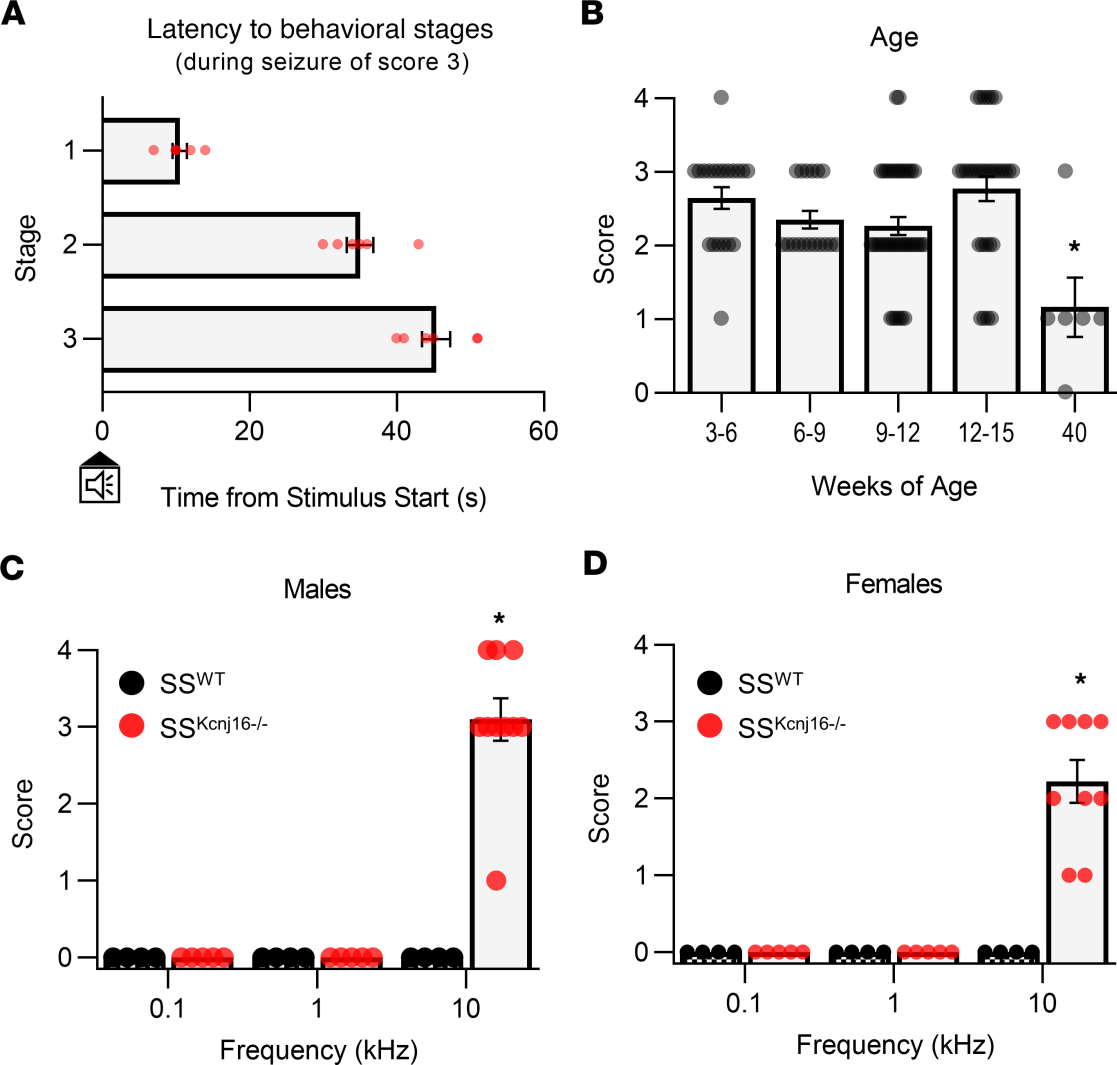
SS^Kcnj16–/–^ rats exhibit audiogenic reflex seizures. (**A**) Latency from the start of the acoustic stimulus (10 kHz) to each of the behavioral stages in seizures that were given a score of 3 (*n* = 6). (**B**) Audiogenic seizure severity scores induced from wean (3 weeks postnatal) to 40 weeks of age (*n* = 4–15 per age group). Scores were decreased at 40 weeks in rats compared with those at 12–15 and 3–6 weeks (*P < 0.05, Kruskal-Wallis ANOVA on ranks with Dunn’s method of multiple comparisons). (**C**) Summary of seizure severity scores in response to acoustic stimuli (0.1, 1, and 10 kHz; 2 minutes each) in male rats (*n* = 4, 4, and 10 for 0.1, 1, and 10 kHz for SS^WT^ rats; *n* = 5, 5, and 10 for 0.1, 1, and 10 kHz for SS^Kcnj16–/–^ rats; *P < 0.001, Kruskal-Wallis ANOVA on ranks). (**D**) Summary of seizure scores in response to acoustic stimuli (0.1, 1, and 10 kHz; 2 minutes each) in female rats (*n* = 4, 4, and 4 for 0.1, 1, and 10 kHz for SS^WT^ rats; *n* = 5, 5, and 9 for 0.1, 1, and 10 kHz for SS^Kcnj16–/–^ rats; *P < 0.001, Kruskal-Wallis ANOVA on ranks). Female rats showed decreased seizure severity compared with male rats (*P* = 0.030, Mann-Whitney rank sum test). Error bars represent mean ± SEM.

In the course of our previous studies evaluating renal effects in SS^Kcnj16–/–^ rats, we fortuitously encountered evidence that indicated the presence of a seizure phenotype. We noted vigorous behavioral seizures occurring in whole cages of SS^Kcnj16–/–^ rats during mixed-frequency ambient noise, such as running water or use of pressurized air. We recognized a behavioral response similar to that described for audiogenic seizures (24–26). Given the association with EAST/SeSAME syndrome, we began to investigate this phenotype and hypothesized that the SS^Kcnj16–/–^ strain could be used as a monogenic model of audiogenic seizures, and we determined the parameters of acoustic stimuli to induce a robust, reproducible response. We found that SS^Kcnj16–/–^ rats consistently and reproducibly exhibited audiogenic seizures when presented with a 10 kHz acoustic stimulus (approximately 75 dB intensity) for a 2-minute duration. This stimulus was sufficient to produce a behavioral seizure response in 93% of SS^Kcnj16–/–^ rats during the first presentation of the tone, which would categorize this novel model as highly susceptible to audiogenic seizures (24). Seizure behaviors typically progressed in a stereotypic manner and were divided into 4 distinct stages, similar to a simplified Racine scale (27) (Table 1). Evoked audiogenic seizures in SS^Kcnj16–/–^ rats began with a short period of immobility (freezing), followed by 1 (stage 1) or 2 (stage 2) phases of vigorous or “wild” running and jumping behaviors, which is characteristic of many other audiogenic seizure models (25). Wild running phases were frequently but not always initiated with a hunched body posture and quick backward movement. Wild running then progressed to clonus (stage 3), in which the animal falls to a recumbent prone position with dorsal flexion and exhibits convulsive spasms and hind limb kicking movements. This was often paired with a brief period of tonicity, thus a seizure of score 3 may be classified as tonic-clonic. If the seizure response terminated at a score of 3, the rat would immediately regain its upright posture and might exhibit vocalizations and/or additional spastic rearing and falling behaviors before entering the postictal phase. If, instead, the seizure advanced to stage/score 4, clonus would be followed by a sustained tonic phase characterized by extension of the fore and hind limbs, apnea, full-body rigidity, and tremors ending with an apparent loss of consciousness without regaining upright posture. Regardless of terminal severity score, the latency from the start of the stimulus to reach behavioral stages 1–3 was about 10, 35, and 45 seconds, respectively (Supplemental Figure 1; supplemental material available online with this article; https://doi.org/10.1172/jci.insight.143251DS1). Latency for each stage during seizures given a score of 3 is depicted in Figure 1A. Representative video recordings portraying seizure behaviors during a seizure score of 4 can be viewed (Supplemental Video 1; rats typically recovered a few minutes after termination of the stimulus; data not shown). We did not observe any incidence of status epilepticus resulting from sound-induced seizures, and none of the audiogenic seizures induced in SS^Kcnj16–/–^ rats acutely led to mortality, as all rats recovered and were ambulatory within approximately 30 minutes.

The audiogenic seizure response exhibited by SS^Kcnj16–/–^ rats was specific for tones in the high frequency audible range. Presentation of 0.1 or 1 kHz tones of the same intensity and duration did not lead to seizures. Seizures induced by the 10 kHz stimulus occurred in both male and female SS^Kcnj16–/–^ rats, but female rats had decreased seizure severity scores compared with male rats (Figure 1, C and D). The most prevalent severity score was a score of 3 in male rats and a score of 2 in female rats (Supplemental Figure 2). Control SS^WT^ rats exhibited no seizure behaviors in response to acoustic stimuli. Audiogenic seizures could reproducibly be evoked in SS^Kcnj16–/–^ rats after weaning (>3 weeks of age) until 40 weeks of age (older ages were not tested). Seizure severity did not appear to be age dependent until 40 weeks of age, when severity declined in response to the acoustic stimulus (Figure 1B). Thus, seizure induction was triggered specifically by high frequency audible stimuli, and this phenotype was apparent as early as 3 weeks of age in SS^Kcnj16–/–^ rats, where seizure severity was greater in male rats compared with female rats.

**Figure 2:**
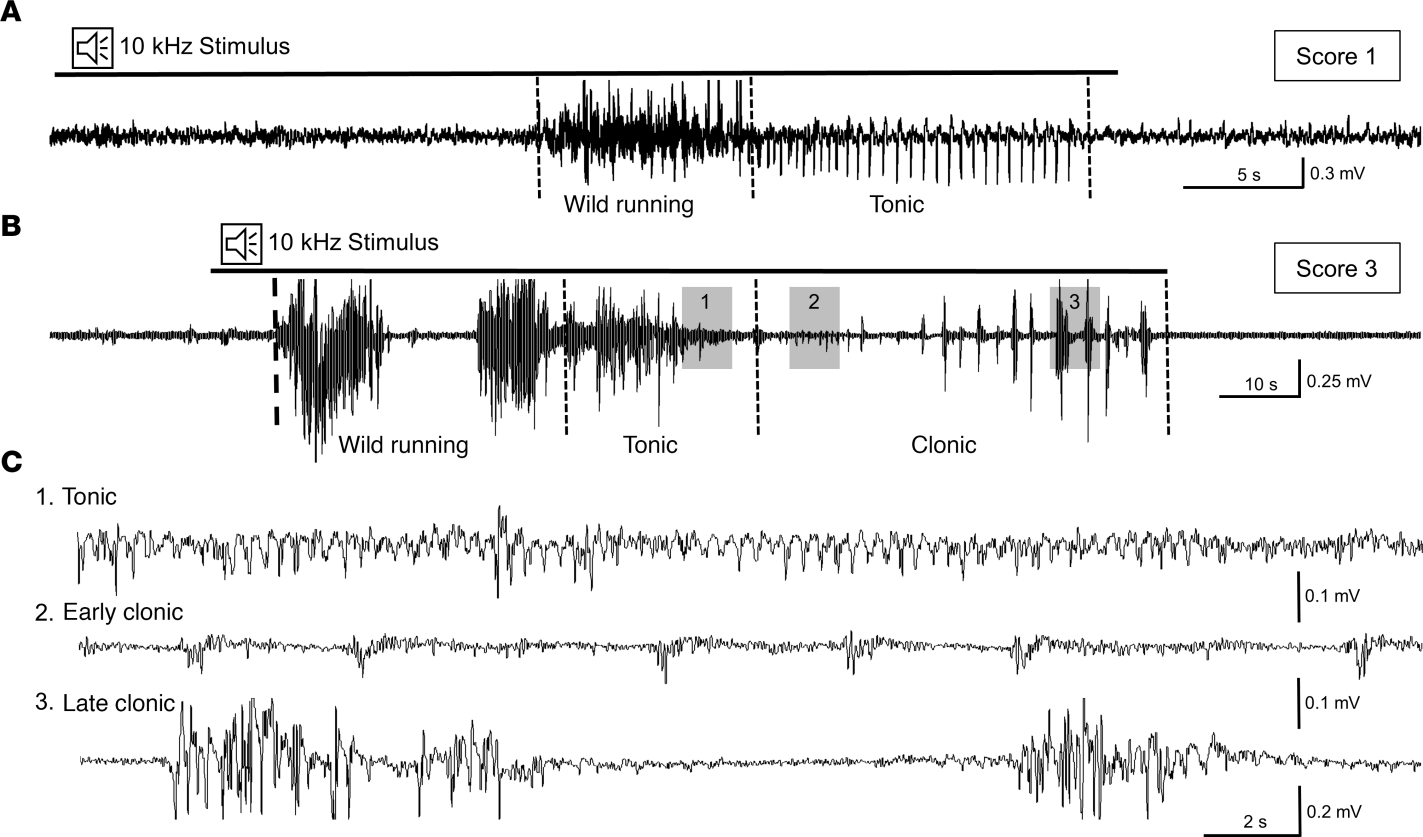
Cortical EEG patterns confirm tonic-clonic audiogenic seizures in SS*^Kcnj16–/–^* rats. The acoustic stimulus (10 kHz, 75 dB, 2-minute duration) induced bilateral tonic-clonic seizures recorded in SS^Kcnj16–/–^ rats (*n* = 4) using implantable radiotelemetry. (**A**) A representative EEG recording for a seizure of score 1 shows a single bout of wild running, followed by a phase of distinct tonic activity. (**B**) A representative recording of a seizure of score 3 is characterized by biphasic wild running, followed by tonic (noted by 1), initial clonic (noted by 2), and late clonic (noted by 3) epileptiform activity. (**C**) Expanded timescale of epileptiform activity highlighted in **B** (1–3, gray).

To confirm the occurrence of cortical electrographic seizures and associate them with corresponding behavioral activity, simultaneous video and electroencephalographic (EEG) recordings were performed in SS^Kcnj16–/–^ rats before, during, and after audiogenic seizure induction (Figure 2). Irregular spiking activity was observed on EEG recordings during episodes of wild running. Movement artifacts during these phases of extreme activity may partially obscure exact patterns of cortical neuronal activity. Sound-evoked wild running developed into well-defined tonic or tonic-clonic electrographic seizures. We found that this could occur even in seizures of scores 1 and 2, where tonic-clonic activity cannot be detected behaviorally. Figure 2A represents an example of an EEG recording during an acoustic stimulus, where wild-running behavior occurred at the end of the 2-minute acoustic stimulation and culminated in tonic seizures with apparent synchronized activity. Figure 2B shows a representative recording of electrographic activity evaluated behaviorally with a score of 3. The EEG recording shows initial biphasic wild running, followed by tonic-clonic activity. The progression of the tonic-clonic activity was reflected in the expanded scale EEG recording (Figure 2C). In addition to the EEG signal, the telemetric EEG recording device (implanted in the abdominal cavity) continuously recorded core body temperatures, which increased approximately 1.4°C by 45 minutes after the termination of the seizures. The magnitude of temperature increase was not found to be dependent on seizure severity and was observed during the postictal period when locomotor activity was reduced (Supplemental Figure 3). These data demonstrated cortical involvement in audiogenic seizures and dramatic seizure-induced increases in body temperatures in the postictal period.

### Inherent CNS hyperexcitability results from Kcnj16 mutation.

**Figure 3:**
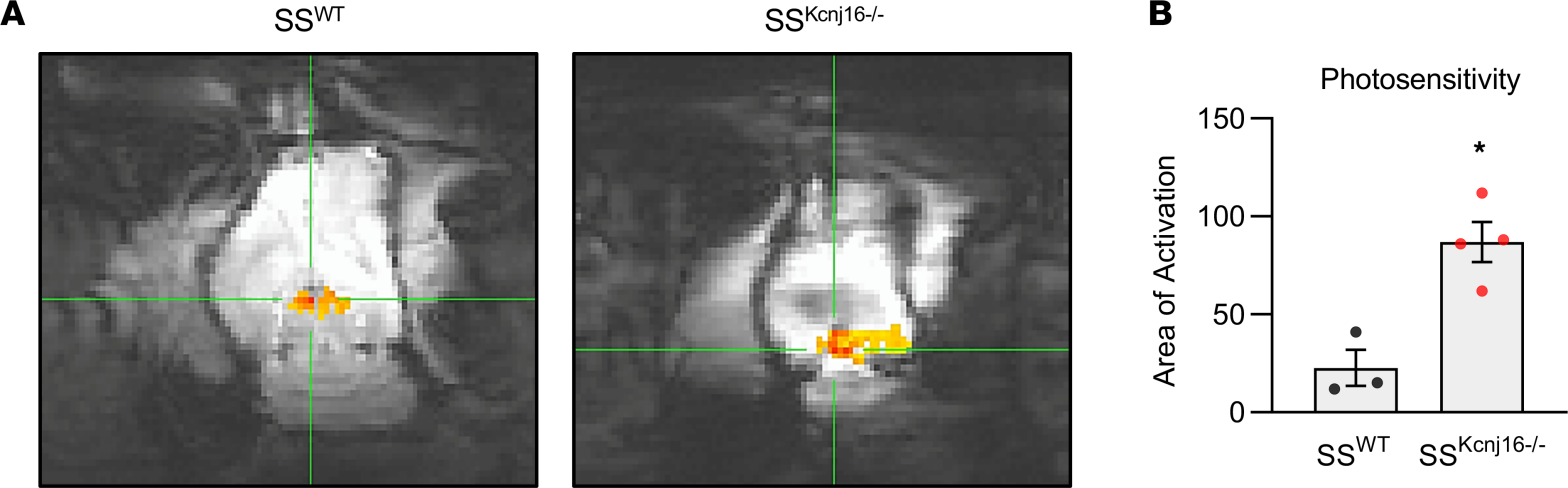
fMRI shows increased photosensitivity in SS^Kcnj16–/–^ rats. (**A**) Representative fMRI images from SS^WT^ and SS^Kcnj16–/–^ rats during the LED photostimulation procedure. The colored areas represent activated voxels (single-shot EPI with 1 mm isotropic resolution, 128 × 128 voxel matrix, 0.3 mm × 0.3 mm). (**B**) Quantitative summary of fMRI light sensitivity assessment in SS^WT^ and SS^Kcnj16–/–^ rats (*n* = 3 and 4; *P = 0.0065, unpaired *t* test). The significance threshold for voxels considered activated above control was 0.01. Error bars represent mean ± SEM.

Functional magnetic resonance imaging (fMRI) was used to provide additional insight into the roles of K_ir_5.1-containing channels in the regulation of brain activity. Since select high-frequency audible tones at modest volumes were sufficient to induce seizures in SS^Kcnj16–/–^ rats, we tested the hypothesis that *Kcnj16* mutants would exhibit features of hyperexcitability in the brain. fMRI revealed no difference in response to somatosensory stimulation (Supplemental Figure 4); however, activation in response to light stimuli was significantly elevated in brains of SS^Kcnj16–/–^ rats, representing increased sensitivity compared with SS^WT^ rats (Figure 3). This enhanced photosensitivity may indicate a predisposition toward neuronal hyperexcitability in the brains lacking K_ir_5.1.

### Audiogenic seizures were prevented with antiepileptic drugs.

**Figure 4:**
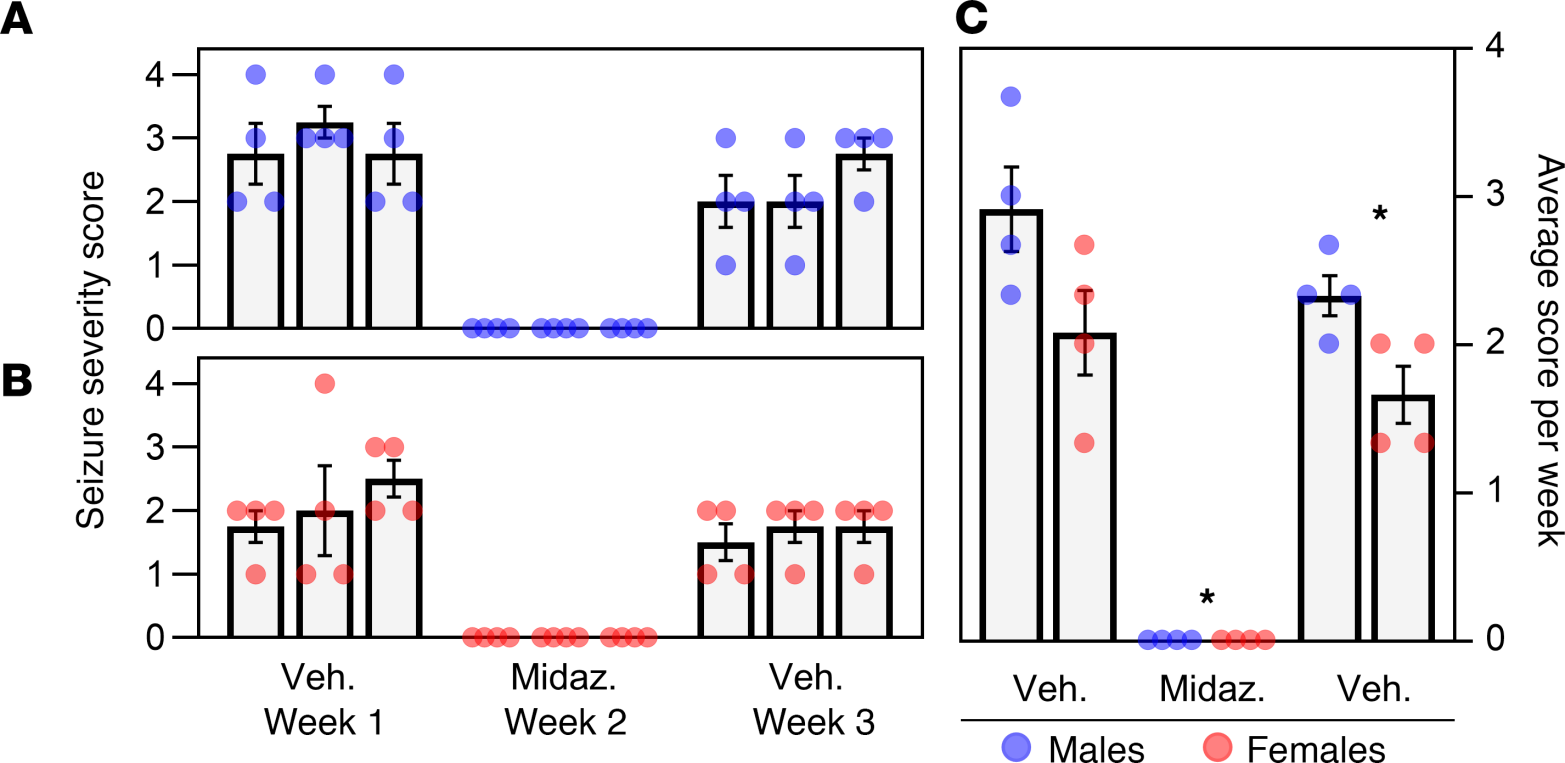
Pretreatment with midazolam prevented audiogenic seizures. (**A**) Male SS^Kcnj16–/–^ rats (*n* = 4, blue) were exposed to the acoustic stimulus 3 times per week for 3 weeks. Rats were pretreated with vehicle (during weeks 1 and 3) or 2.5 mg/kg i.p. midazolam (during week 2) 1 hour before each acoustic stimulation. Data points represent individual scores, bars represent the group mean severity per day, and error bars represent mean ± SEM. (**B**) The protocol described in **A** was repeated with female SS^Kcnj16–/–^ rats of the same age (*n* = 4, red). (**C**) Plot representing an analysis of average seizure severity achieved per week in male (blue) and female (red) SS^Kcnj16–/–^ rats summarized from data shown in **A** and **B**. Data points represent the average severity score per animal per week; bars represent the group average per week; and error bars represent mean ± SEM. Midazolam pretreatment entirely prevented seizures in both male and female SS^Kcnj16–/–^ rats during week 2 (*P < 0.001). Seizure responses were restored with vehicle treatment during week 3, but severity was significantly lower in both sexes (*P* = 0.009). Male rats had more severe seizures than female rats during weeks 1 and 3 (*P* = 0.044). Statistical analysis performed was a 2-way repeated-measures ANOVA with Holm-Sidak method of multiple comparisons. Asterisks indicate a significant difference in seizure severity from week 1.

To evaluate whether benzodiazepine antiepileptic drugs (AEDs) could prevent the SS^Kcnj16–/–^ audiogenic seizure response in vivo, we administered saline (vehicle), midazolam (2.5 mg/kg i.p.) or diazepam (2.5 mg/kg i.p.) before (>1 hour) exposure to the acoustic stimulus. Pretreatment of male and female SS^Kcnj16–/–^ rats with vehicle injections before each of the 3 acoustic stimulations during week 1 established a baseline seizure severity for each rat. Midazolam pretreatment during week 2 prevented all seizure responses in male and female rats across all 3 studies, but seizure responses returned after vehicle treatment during week 3 (Figure 4, A and B). Average seizure severity scores for each rat during each week are presented in Figure 4C, which showed that while all seizure responses returned during week 3, the seizure severity scores were reduced for male and female rats compared with week 1, showing no evidence of rebound hyperexcitability after midazolam treatment. Additionally, male rats demonstrated increased seizure severity scores relative to those of female rats during both weeks 1 and 3, consistent with previous observations (Figure 1, C and D). Pretreatment with another benzodiazepine diazepam was also effective in preventing audiogenic seizures in male and female SS^Kcnj16–/–^ rats (Supplemental Figure 5). Thus, frontline AEDs were effective in preventing audiogenic seizures in this model but had no lasting effects within 1 week of withdrawing treatments.

### Repeated seizures (3 days) prolonged postictal recovery time and decreased locomotor behavior.

**Figure 5:**
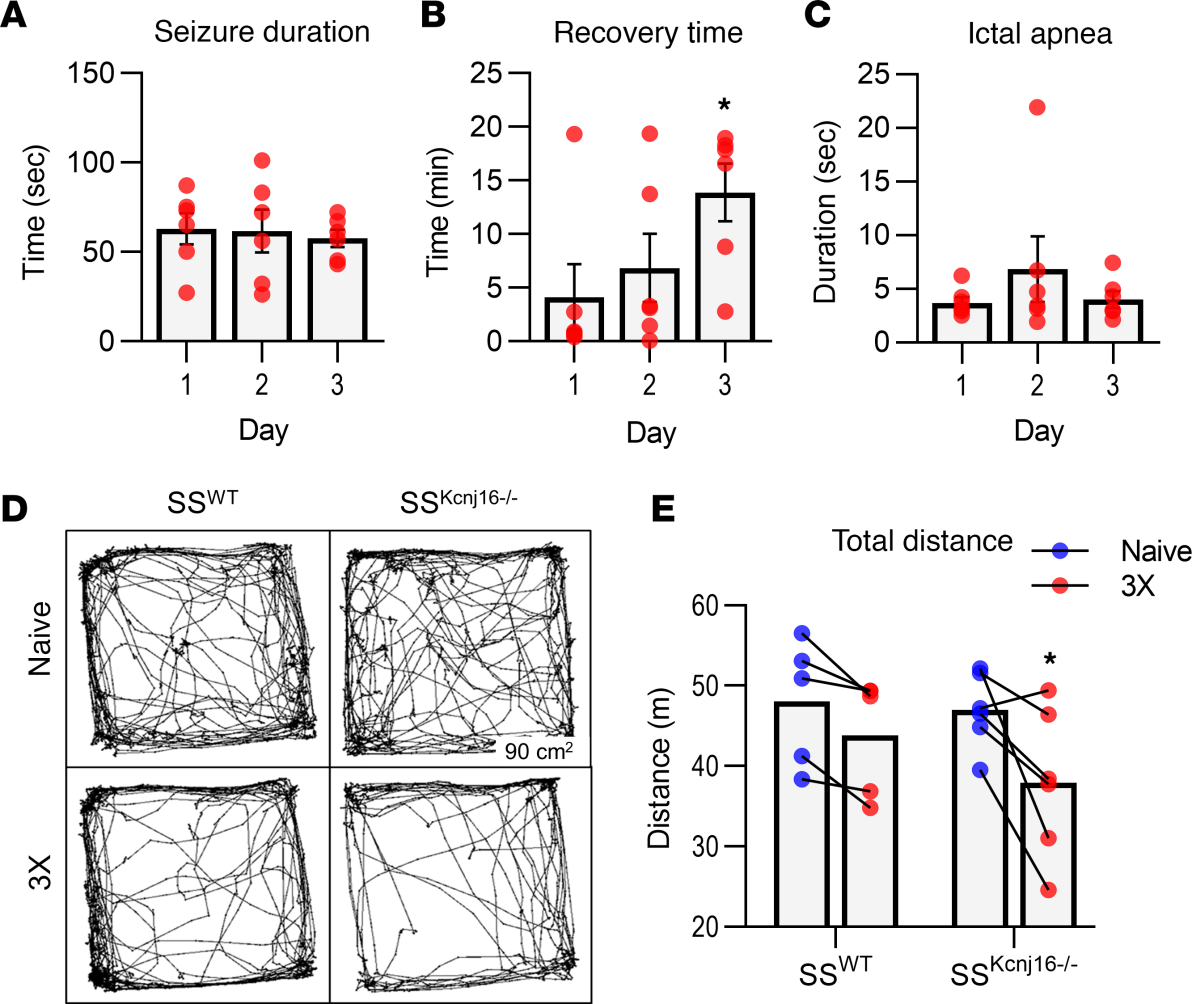
Effects of 3 seizure exposures on SS^Kcnj16–/–^ rat behavior. SS^Kcnj16–/–^ and SS^WT^ rats were exposed to the acoustic stimulus once per day for 3 days. (**A**) Seizure duration in seconds for *n* = 6 SS^Kcnj16–/–^ rats plotted for each of the 3 days. (**B**) Recovery time (minutes) after a seizure, determined by the time to resume normal ambulatory behaviors, plotted for each day (*n* = 6). Recovery time was significantly increased on day 3 (*P = 0.016 comparing days 1 and 3, 1-way repeated-measures ANOVA with Holm-Sidak multiple comparisons). (**C**) Duration of the apnea that occurs during the tonic phase of the seizure did not change over the 3 days (*n* = 6). (**D**) Representative open-field tests with locomotion mapped (90 × 90 cm enclosure) before and after 3 exposures to the acoustic stimulus. (**E**) Total distance traveled during the 20-minute test period significantly decreased in SS^Kcnj16–/–^ rats (*n* = 6) after 3 seizures (*P = 0.007, 2-way repeated-measures ANOVA with Holm-Sidak multiple comparisons) but was unchanged in SS^WT^ rats after 3 exposures to the same acoustic stimulus (*n* = 5; *P* = 0.171). The connecting lines designate paired values to show change from before (blue data points) to after (red data points) completing the 3× acoustic stimulation protocol.

To determine if repeated seizures caused neurobehavioral deficits, SS^Kcnj16–/–^ and SS^WT^ rats were exposed to the acoustic stimulus once daily for 3 days (3× seizure protocol). SS^Kcnj16–/–^ rats did not show increased total seizure duration or increased duration of the ictal apnea occurring during tonus (Figure 5, A and C). However, it took longer for rats to recover (resume normal ambulatory behaviors) on day 3 compared with days 1 and 2 of the 3× seizure protocol (Figure 5B). In addition, data from open-field tests revealed a decrease in total distance travelled after completing the 3× seizure protocol in SS^Kcnj16–/–^ rats but not SS^WT^ rats (Figure 5, D and E). However, we found no change in anxiety-related behaviors (represented by the amount of time spent in the center versus periphery of the open field) following the 3× seizure protocol (Supplemental Figure 6). A modified Irwin screen (assessing various physical attributes, behavioral parameters, and reflexes) was conducted before and after the 3× protocol, where SS^Kcnj16–/–^ rats exhibited greater changes in Irwin screen parameters, including physical state, anxiety behaviors, and activity after 3 days of seizures compared with SS^WT^ controls exposed to the sound stimulus (Supplemental Figure 7).

### High mortality rates following repeated seizures (10 days).

**Figure 6:**
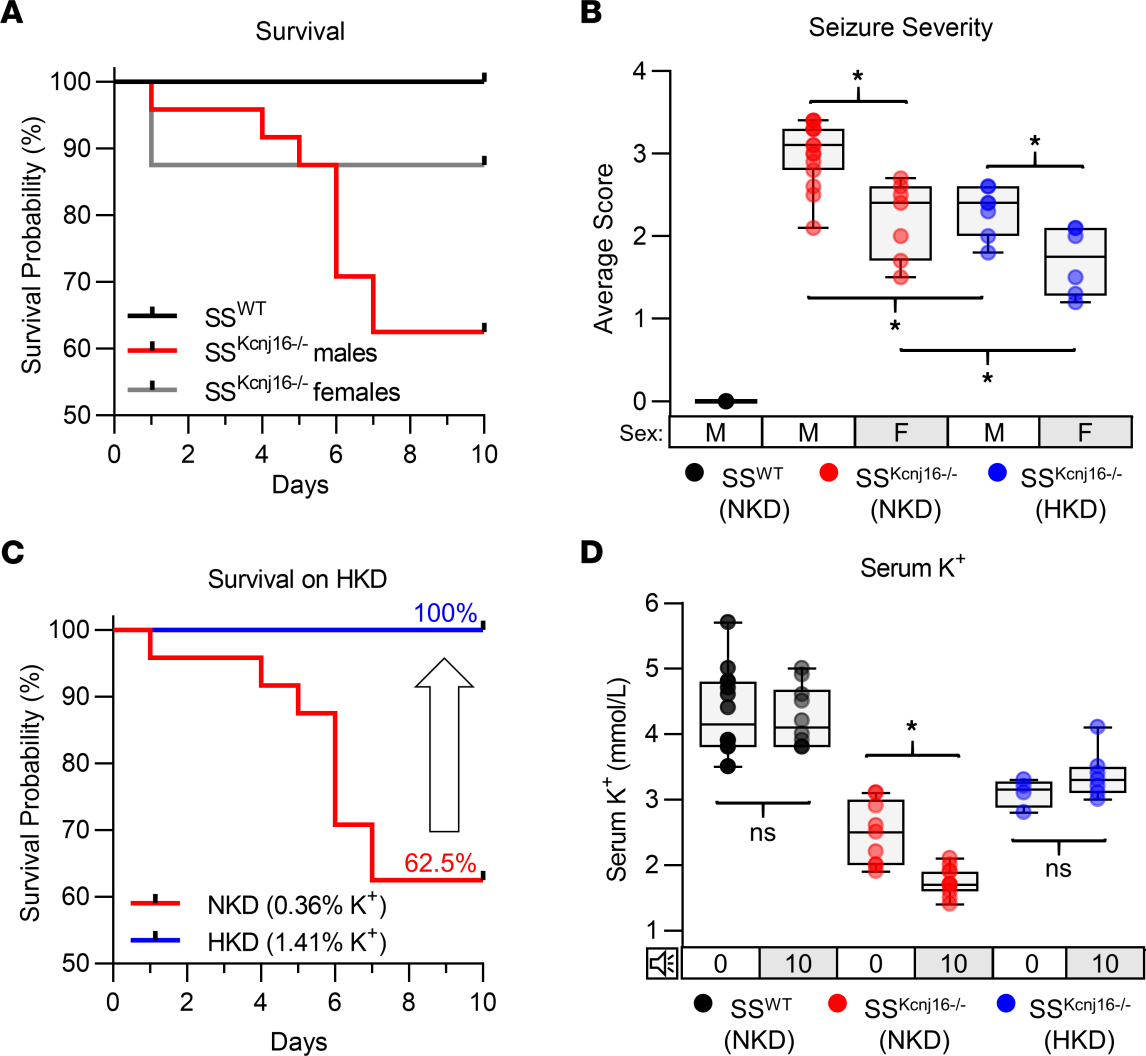
SS^Kcnj16–/–^ rats experience mortalities during 10 days of repeated seizure exposure. (**A**) Kaplan-Meier survival analysis of SS^WT^ and SS^Kcnj16–/–^ rats during once-daily exposure to the 10 kHz acoustic tone. Repeated seizure induction (once daily for 10 days) resulted in mortalities in 37.5% of male and 12.5% female SS^Kcnj16–/–^ rats (*n* = 24 and 8, respectively). SS^WT^ rats (*n* = 12) subjected to the same 10× protocol did not exhibit any mortalities. The survival curves depicted are significantly different (*P* = 0.0339, Mantel-Cox log-rank test). (**B**) Average seizure severity score (scores averaged over the 10 days for each animal) in male and female SS^Kcnj16–/–^ rats during the 10× protocol compared between rats fed a normal K^+^ diet (NKD, 0.36% K^+^) and a high-K^+^ diet (HKD, 1.41% K^+^). Dietary K^+^ supplementation reduced average seizure severity in male and female SS^Kcnj16–/–^ rats but did not alter seizure incidence (*P < 0.001, 2-way ANOVA with Holm-Sidak method of multiple comparisons). Female SS^Kcnj16–/–^ rats were found to have lower average severity than male rats on both diets. (**C**) Kaplan-Meier survival curve showing that dietary K^+^ supplementation prevented mortalities from repeated seizure exposure in SS^Kcnj16–/–^ rats (*n* = 10 and 6 for male and female rats, *P* = 0.0145). (**D**) Serum K^+^ was measured 24 hours after completion of the 10-day stimulation protocol (labeled “0”) and compared with values measured in age- and diet-matched naive rats (labeled “10”). 10× repeated seizure exposure in SS^Kcnj16–/–^ rats on the NKD resulted in decreased serum K^+^ compared with naive that in SS^Kcnj16–/–^ rats (*P < 0.001, 2-way ANOVA with Holm-Sidak method of multiple comparisons) (*n* = 13 and 10; 9 and 11; and 4 and 7 for control and 10 exposures in SS^WT^ on NKD [black], SS^Kcnj16–/–^ on NKD [red], and SS^Kcnj16–/–^ rats on HKD [blue], correspondingly).

To evaluate whether the neurological phenotypes observed in SS^Kcnj16–/–^ rats would be magnified by additional days of seizure induction, audiogenic seizures were induced once daily for up to 10 days (10× seizure protocol). Throughout the 10× seizure protocol we noted mortality of 37.5% of male (9 of 24 total) and 12.5% of female (1 of 8 total) SS^Kcnj16–/–^ rats (Figure 6A). All mortalities occurred well after the rat had behaviorally recovered from the seizure (resumed normal ambulation and grooming behaviors and had been returned to its home cage environment) and were independent of seizure severity score. The time frame for these deaths was variable; it occurred between 2 and 12 hours after a seizure was induced and was therefore not likely a direct result of the seizure events. In contrast, all control SS^WT^ rats (which underwent the same daily sound exposures) did not exhibit seizures and survived the 10× protocol (Figure 6A). A modified Irwin screen performed on surviving SS^Kcnj16–/–^ rats before and after the 10× seizure protocol showed greater changes in neurobehavioral parameters, including altered physical state, anxiety-like behaviors, and activity compared with SS^Kcnj16–/–^ rats that underwent the 3× seizure protocol (Supplemental Figure 8). We did not observe any correlation between behavioral or EEG seizure severity and the number of audiogenic seizure repetitions, suggesting an absence of kindling or priming processes derived from recurring acoustic stimulation (Supplemental Figure 9).

### Dietary K^+^ supplementation reduced seizure severity and prevented mortalities with repeated seizures.

Hypokalemia is an inherent feature in SS^Kcnj16–/–^ rats, which leads to 100% mortality when animals are fed a high-sodium diet. However, salt-induced mortality can be prevented in this model by a high-K^+^ dietary supplement (18). To test if the mortalities triggered by repeated seizures correlate with blood K^+^ levels, rats were fed (from weaning) either a normal- or a high-K^+^ diet (NKD or HKD, 0.36% and 1.41% KCl, respectively). Dietary K^+^ supplementation did not prevent audiogenic seizures but reduced average seizure severity in both male and female SS^Kcnj16–/–^ rats (Figure 6B). Importantly, SS^Kcnj16–/–^ rats fed the HKD before and throughout the 10× seizure protocol showed a 100% survival rate (Figure 6C). Repeated seizures in the 10× seizure protocol led to an exacerbation of the hypokalemia in SS^Kcnj16–/–^ rats fed the NKD, which was prevented in rats fed the HKD (Figure 6D). The HKD did not alter changes in body temperature in the postictal period (Supplemental Figure 10).

## Discussion

K_ir_ channels have long been considered essential contributors to cellular excitability and K^+^ homeostasis in many types of cells. Among the multiple K_ir_ family members, K_ir_5.1 has received less attention, ostensibly because existing data suggest K_ir_5.1 subunits cannot form a functional homotetrameric channel in vivo. However, the inclusion of K_ir_5.1 subunits in heterotetrameric K_ir_ channels significantly modifies the channels’ biophysical properties in several ways, including increasing conductance and pH sensitivity. Thus K_ir_5.1 subunits compose unique channels distinct from other homomeric K_ir_ channels, which could be essential contributors to many physiological functions. K_ir_5.1 is primarily expressed in the kidney and brain, where its roles in fundamental biological processes are beginning to be unravelled, although its contributions to human pathologies remain unclear. We recently showed that mutations of *Kcnj16* alter K^+^ homeostasis and can be protective against salt-induced hypertension (18). In addition, our previous work indicates that K_ir_5.1 expressed in brainstem respiratory nuclei contributes to acute pH homeostasis via the ventilatory chemoreflex (20). Studies in *Kcnj16*-knockout mice have also reported a reduction in central chemosensitivity and/or impaired chemoreflexes (28, 29) and other similar phenotypes, including metabolic acidosis, hypokalemia, and low body weight (30). However, the Kcnj16^–/–^ mice showed no difference in blood pressure when compared with WT mice, while the SS^Kcnj16–/–^ rats exhibited low blood pressure and protection against salt-induced hypertension compared with SS^WT^ rats. This difference is very likely due to the hypertensive genetic background of the Dahl SS rat. In addition, both mouse and rat models have deficits in central chemosensitivity or chemoreflexes indicating at least some level of CNS pathology (20, 28, 29). However, it is unclear whether neuronal excitability or seizure susceptibility have been assessed in the Kcnj16^–/–^ mice. We reasoned that loss of function in K_ir_5.1 would not only interfere with respiratory control and pH homeostasis, but would result in widespread dysfunction in the CNS through increased neuronal excitability. Herein, we used SS^Kcnj16–/–^ rats to evaluate the neurological role of K_ir_5.1 channels and the potential pathophysiological consequences of its loss of function.

The sum of the data confirms our hypothesis. SS^Kcnj16–/–^ rats showed inherently increased responses to light stimulation via fMRI and were found to be highly susceptible to sound-induced, generalized tonic-clonic seizures. Thus, the data suggest that a loss-of-function mutation in K_ir_5.1 may cause destabilization of the resting membrane potential and subsequent neuronal hyperexcitability, leading to gross neurologic dysfunction. Audiogenic seizures in SS^Kcnj16–/–^ rats were (a) readily induced in young and aged rats, (b) sound frequency dependent, and (c) confirmed both behaviorally and with EEG recordings. These audiogenic seizures were highly repeatable and could be blocked entirely by benzodiazepines. This robust seizure phenotype strongly indicates that K_ir_5.1 plays a critical, nonredundant role in modulating neuronal excitability in the CNS. It also suggests *Kcnj16* as a potential candidate in the study of human epilepsies and/or seizure disorders.

Patients with drug-resistant (refractory) epilepsies are faced with diminished quality of life and a 24-fold increased risk of spontaneous seizure-related mortality (19). SUDEP represents a major cause of death in this patient cohort and affects patients of all ages (31). The primary cause of death in SUDEP is unclear but likely results from cardiorespiratory suppression either immediately following a seizure or as much as 2 hours into the postictal period (32). Repeated seizures in SS^Kcnj16–/–^ rats lead to increased mortality, which occurred after seizure induction (2–12 hours). Interestingly, our data suggest that dietary K^+^ supplementation may significantly reduce mortality and provide a reduction in severity (but not incidence) of acoustically induced seizures. Furthermore, we showed that tonic-clonic seizures induced prolonged ictal apneas in this model, consistent with data presented in the MORTEMUS study (33). While additional characterization of the effects of individual and repeated seizures in this model is required, it may represent a useful tool in filling existing gaps in knowledge as to how repeated seizures negatively affect vital cardiorespiratory mechanisms and lead to increased risk of SUDEP — a goal likely only to be achieved using appropriate animal models (34). Understanding the potential contribution of lesser-studied ion channel genes, such as *Kcnj16*, to human epilepsy will unlock opportunities to uncover genetic contributors to idiopathic epilepsy and bring forth new treatment strategies for those resistant to current AEDs.

Our findings strongly indicate that loss of function of K_ir_5.1 likely produces a predisposition for hyperexcitability in the brain, but it remains unclear precisely which brain areas and cell types are most affected by the mutation. However, the origins of audiogenic seizures are largely thought to arise from subcortical brain regions, including the brainstem (35), and behavioral patterns in SS^Kcnj16–/–^ rats indicate generalized rather than partial seizures, as behaviors are exhibited symmetrically in both sides of the body. The presence of audiogenic seizures in SS^Kcnj16–/–^ rats combined with our previous findings of deficits in CNS-driven respiratory chemoreflexes strongly support a major role for K_ir_5.1 in vital brainstem functions (20). Moreover, K_ir_5.1 has been shown to be more highly expressed in the brainstem compared with the cortex (36). We predict that seizures in SS^Kcnj16–/–^ rats likely have brainstem involvement, but more work needs to be done to identify precisely where seizures originate as well as other structures involved. Future studies will also be required to gain mechanistic insight into the neurological role of K_ir_5.1 on a cellular level. This is especially true as expression of K_ir_5.1 across CNS cell types has not been comprehensively established.

The SS^Kcnj16–/–^ rat represents a unique model of audiogenic seizures in several ways, including our finding that repeated seizures produce enhanced mortality in a rat model with a known genetic mutation. This model allows for tightly controlled experiments with repeated seizure administration to be performed while limiting confounding factors in a species that allows for a wide array of physiological measurements. The 10 kHz acoustic stimulus readily and reliably produces a generalized tonic-clonic seizure response, allowing for control over the desired number of seizure exposures. Sound-induced seizures are especially advantageous because they do not require pharmacological or painful stimuli or surgical interventions in the CNS that may confound physiological data (26). The acoustic stimulus does not even require high amplitudes to induce robust seizures (whereas some audiogenic models require 115–120 dB) (24, 37). Furthermore, the SS^Kcnj16–/–^ model would be categorized as highly susceptible to audiogenic seizures, as a single presentation of the acoustic stimulus produced robust seizure responses in 93% of rats. The SS^Kcnj16–/–^ rat model is also innately susceptible to audiogenic seizures and does not require kindling or priming to evoke seizures. Among the audiogenic models, the SS^Kcnj16–/–^ rat phenocopies a monogenic disorder with a single, known dysfunctional protein, in contrast to the models without identified heritability or with polygenic origins. This factor is important for drug development; for instance, the efficacy of benzodiazepines in this model indicates that GABA-sensitive pathways may counterbalance K_ir_5.1-dependent seizures. Although the genetic background of this model, the Dahl SS rat, is most commonly used as a model of hypertension and renal injury, SS^Kcnj16–/–^ rats do not exhibit either of these phenotypes (18). The Dahl SS rat is a long-established inbred strain that has been subject to extensive genetic and genomic study (this colony has been maintained on site at Medical College of Wisconsin since 1991). Generations of inbreeding has fixed the genetic loci providing valuable genetic uniformity, and the absence of residual heterozygosity has been confirmed (38). In addition, the Dahl SS (originally derived from Sprague-Dawley) genetic background may provide an alternative to the panel of the Wistar-based strains (KM, AS-Wistar, WAR) that develop audiogenic seizures (39) and the genetically epilepsy-prone Sprague-Dawley rat (40). Interestingly, unlike in SS^Kcnj16–/–^ rats, benzodiazepine treatment does not prevent seizures in KM rats, although it reduces catalepsy. Additionally, KM rats exhibit high endogenous anxiety in elevated plus maze (39), whereas naive SS^Kcnj16–/–^ rats demonstrate more stable psychomotor behavior, which may be beneficial if drug testing considers differences in psychic profile of the strain.

A role for K_ir_5.1 in epilepsy and/or seizure disorders has not been previously addressed, despite the strong evidence linking the related protein, K_ir_4.1 (encoded by *Kcnj10*), to seizure disorders (9, 10, 41). Although K_ir_4.1 and K_ir_5.1 proteins coassemble to make a functional heterotetrameric channel in the kidney; the composition, location, and function of K_ir_5.1-containing channels are less clear in the brain. The complementary but nonredundant functions of K_ir_4.1 and K_ir_5.1 suggest that K_ir_5.1 may offer a promising target for research to further our understanding of genetic factors in epilepsy. Our work confirms this and supports K_ir_5.1 as viable candidate for pharmacological targeting and clinical therapeutics, which is especially promising considering recent progress in the development of specific small-molecule K_ir_ channel modulators (42, 43). We present the SS^Kcnj16–/–^ strain as a highly reproducible, easy-to-use genetic (versus electrically/chemically inducible) model of reflex (versus spontaneous) generalized (versus partial) seizures suitable for AED screening.

## Methods

### Animals.

SS^Kcnj16–/–^ and SS^WT^ rats were acquired from colonies sustained at the Medical College of Wisconsin. Methods for the generation of SS^Kcnj16–/–^ rats (SS-Kcnj16^em1Mcwi^) have been previously published (18). Rats were housed in controlled environmental conditions under a 12-hour-light/dark cycle with food and water provided ad libitum. Unless otherwise indicated, rats were studied at approximately 10 weeks of age. Animals were fed a standard diet, ad libitum, consisting of either a purified AIN-76A food (Dyets Inc., D113755) (NKD; 0.4% NaCl and 0.36% K^+^), or a diet containing supplemental K^+^ (Dyets Inc., D113521) (HKD; 0.4% NaCl or 0.4% NaCl and 1.41% K^+^). K^+^ content in both diets are in a healthy range for the nutrition requirements of rats (44).

### Seizure induction and behavioral scoring.

Seizure induction occurred in isolation from other animals in a custom-built plexiglass whole-body plethysmograph used for breathing measurements during the seizure protocol (45, 46). In all cases, rats were removed from their home cage and given 20 minutes to acclimate to the experimental chamber before acoustic stimulation began. The stimulus for seizure induction was a 10 kHz audio frequency produced by a function generator (GW Instek model GFG-8020H) and delivered through a 50 Ω speaker (Visaton model k50wp) positioned about 5 inches above the rat. The stimulus was presented for a 2-minute duration at about a 75 dB amplitude. Presentation of the stimulus was repeated 1 time in the rare case that a behavioral seizure response was not produced (only about 7% required repeat stimulation). Seizure behaviors were video recorded, and seizure severity was scored based on a modified Racine scale optimized for the specific progression of behaviors consistently observed in these animals. Modification of the Racine scale has been shown to be required in other seizure models (27, 47). For each seizure, a final seizure severity score of 0–4 was determined by the ultimate behavioral stage reached during the seizure (Table 1): score 0, no distinctive seizure behaviors observed; score 1, 1 bout of wild running (uniphasic); score 2, 2 bouts of wild running (biphasic) or wild running continuing for an extended duration; score 3, wild running, followed by clonic or tonic-clonic behaviors and upright posture regained immediately after seizure; and score 4, wild running followed by tonic-clonic behaviors ending with a sustained period of tonic extension and apparent loss of consciousness and upright posture regained after about 5 minutes of recovery.

For repeated seizure exposures, the same seizure induction protocol was used and induction was repeated once daily for either 3 or 10 days for each rat. SS^WT^ control rats did not exhibit any seizure responses but underwent the same protocol, including a 20-minute acclimation period, 2-minute acoustic stimulation, 20-minute recovery period, and behavioral scoring.

### EEG telemetry.

To monitor EEG in freely moving rats, rats underwent surgery to implant electrodes and a transmitter for the telemetric recording system (Data Science International). Briefly, rats were deeply anesthetized with isoflurane (5% induction, 2.5% maintenance in oxygen at 1 L/min). The fur on the head and abdomen was shaved and skin was disinfected with betadine scrub and 70% ethanol. Ear bars (with 5% lidocaine gel local anesthetic) were used to secure the rat in position on the stereotaxic instrument (Kopf model 693 no. 311006R), and the surgical platform was warmed to maintain body temperature. A precision drill (Foredom K.1070 High Speed Rotary Micromotor) mounted on the stereotaxic frame was used to precisely drill bilateral burr holes (0.9 mm, FST no. 19007-09) over the parietal cortex (2 mm caudal to bregma, 2.5 mm lateral to midline) and cerebellum (1.5 mm caudal to lambda, 2 mm lateral from the midline) for reference electrodes. The transmitter (TL11M2-F40-EET) was placed in the abdominal cavity, and electrodes (2 EEG electrodes paired with 2 reference electrodes) were tunneled under the skin to be inserted epidurally into burr holes and secured with anchoring screws (1.9 mm, FST, 19010-00) and dental cement (Integrity, 666320). Buprenorphine for analgesia (slow release 0.5 mg/kg s.c.) and enrofloxacin (10 mg/kg s.c.) were administered i.p. After surgery, rats continued to receive supplemental warmth and were monitored until conscious and ambulatory before being housed individually with additional enrofloxacin administered in the water (0.1 mg/mL ad libitum). Animals were allowed at least a 10-day recovery period after surgery before beginning seizure induction and were monitored daily for locomotion, breathing, swelling, coat, and surgical site. Cages were positioned on top of receiver pads to enable continuous unrestrained EEG recording. Telemetry data were acquired using DSI Dataquest software. EEG signal was sampled at 1000 Hz, and data for temperature, activity, and signal strength were sampled at 1 Hz. Electrographic seizures were defined as the emergence of rhythmic spikes with a frequency of 1 Hz or more lasting at least 10 seconds. EEG data were analyzed and interpreted offline using pCLAMP 10.6 software (Molecular Devices Corp).

### fMRI.

A 9.4T Bruker AVANCE MRI scanner was used to collect MRI on a subset of rats. An anatomical image was acquired with a rapid acquisition with relaxation enhancement sequence (matrix size 256 × 256, TE = 56 milliseconds, TR = 5000 milliseconds). For the fMRI acquisition, gradient echoes (single-shot EPI, TE = 8.75 milliseconds, TR = 2 seconds, 90-degree flip angle, matrix size 96 × 96, number of repetitions = 110, 10 contiguous interleaved 1 mm slices, acquisition time of 3 minutes 40 seconds) were acquired during a block design stimulus paradigm. The fMRI task data were processed using the Analysis of Functional NeuroImages (AFNI) software (48). The fMRI signal was modeled using a boxcar function as a regressor and timed on the stimulus, and activation was determined by an F test with a *P* value threshold of 0.01 using AFNI. Voxels with significance greater than the threshold were considered activated.

Animals aged 8–10 weeks were subjected to fMRI study once or twice per week. Animals were anesthetized with isoflurane (approximately 5% for induction and 0.5%–3% for maintenance) for the duration of the procedure. For complex brain function studies, dexmedetomidine infusion anesthetic was used as a supplement through a tail IV infusion. While anesthetized, animals were placed on a heated surface to maintain body temperature. Differences in neural activity were detected in response to light-induced activation stimuli or electrical stimulation of the forepaw. Photostimulation to activate the visual cortex occurred via flashes presented to the eyes by light-emitting diodes (LEDs) of 480 nm wavelength. The LEDs were bilaterally positioned 2–3 cm in front of the eye (lubricated with Aquatears [Ciplamed]). Forepaw electrical stimulation of the somatosensory cortex was delivered through needle electrodes inserted s.c. into the webspace between forepaw digits 2–3 and 3–5. Electrical stimulation was produced by a Grass Telefactor constant current stimulation (S48) at 2 mA, 3 ms, 3–10 Hz for 10 seconds.

### Administration of AEDs.

AED treatment consisted of either midazolam (AKORN, NDC: 17476-524-10) or diazepam (MilliporeSigma, D0899). Acoustic stimulations occurred as described above in a whole-body plethysmograph to allow breathing to be monitored. Seizure severity was scored behaviorally as described previously. In the midazolam protocol, male and female SS^Kcnj16–/–^ rats were exposed to the acoustic stimulus 3 times per week for 3 weeks. Approximately 1 hour before acoustic stimulation, rats were given an i.p. injection of vehicle (0.9% saline administered during weeks 1 and 3) or 2.5 mg/kg midazolam during week 2. In the diazepam protocol, male and female SS^Kcnj16–/–^ rats were presented with the acoustic stimulus once per week for 3 weeks. Approximately 1 hour before acoustic stimulation, rats were given an i.p. injection of vehicle (0.9% saline administered during weeks 1 and 3) or 2.5 mg/kg diazepam during week 2.

### Behavioral testing.

All behavioral assessments were performed at the Medical College of Wisconsin’s Neuroscience Research Center with protocols and training from the rodent behavior core. Behavior tests were performed on the same rats before and after repeated seizure exposure (either 3× or 10×). An open-field test was used to assess anxiety and general locomotion in SS^Kcnj16–/–^ and SS^WT^ rats. Each animal was placed in a square open-top plexiglass enclosure (90 × 90 cm) and allowed to explore for 20 minutes without an observer present in the room. Video camera and tracking software (IC Capture camera control software) was used to record the enclosure from above, chart the animal’s locomotion, and determine the proportion of time the animal was positioned along the perimeter of the apparatus compared with time spent in the center. The enclosure base was dark gray in color to provide adequate contrast with the white rats to allow the software to accurately track the rats position. The enclosure was cleaned thoroughly with 70% ethanol before each test. Individual video recordings from open-field tests were analyzed using Ethowatcher software freely available for noncommercial use from the Laboratory of Bioengineering of the Institute of Biomedical Engineering and the Laboratory of Comparative Neurophysiology at the Federal University of Santa Catarina in Brazil (49). A modified Irwin screen was used as a systematic measure of general and neurological health. Parameters scored include general appearance, muscle tone, hearing, reflexes, temperature, body weight, novel transfer behavior, and more. See Supplemental Table 1 for the Irwin screen scoring rubric used.

### Statistics.

Much of the data collected was analyzed for statistical significance using 1- or 2-way ANOVAs with appropriate normality and equal variance pretests. For example, weekly average seizure severity in AED administration studies were tested for normality (Shapiro-Wilk’s test) and equal variance, then compared using a 2-way repeated-measures ANOVA with sex and treatment as factors, followed by Holm-Sidak multiple comparisons. Behavioral data (open-field test) required a 2-way repeated-measure ANOVA with strain and treatment as factors. Because seizure severity was scored on an ordinal scale of 0–4, analyses comparing raw seizure scores required nonparametric statistical tests to accommodate this ordinal data. Data were tested for normality (Shapiro-Wilk’s test) and equal variance before being subjected to the Mann-Whitney rank sum test. For ordinal data requiring repeated-measures analysis, a Friedman 2-way repeated-measures ANOVA on ranks was performed, followed by Tukey’s test for multiple comparisons. For all analyses, *P* values of less than 0.05 were considered significant.

### Study approval.

All procedures and protocols were reviewed and approved by the Medical College of Wisconsin Institutional Animal Care and Use Committee before experiments began.

## Author contributions

ADM, OP, MRH, and AS conceived the study. ADM, OP, EI, VL, TSP, and MRH provided investigation. ADM wrote the original draft of the manuscript. ADM, OP, MRH, and AS reviewed and edited the manuscript. ADM, OP, EI, and PSL analyzed data. AS, OP, and MRH provided resources. AS supervised the study. All authors approved the final version of the manuscript.

## Supplementary Material

Supplemental data

Supplemental Video 1

## Figures and Tables

**Table 1:**
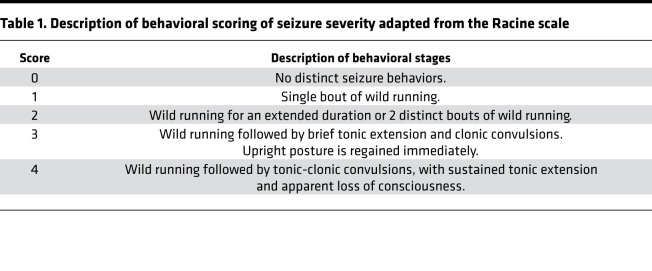
Description of behavioral scoring of seizure severity adapted from the Racine scale

## Version 1 11/24/2020

In-Press Preview

## Version 2 01/11/2021

Electronic publication
