# Supplementary material for: Kcnj16 knockout produces audiogenic seizures in the Dahl salt-sensitive rat
Source: JCI Insight. 2021 Jan 11;6(1):e143251. doi: 10.1172/jci.insight.143251 (PMC7821607; doi:10.1172/jci.insight.143251)
Supplement: Supplemental data [file jciinsight-6-143251-s118.pdf]

Supplemental materials

Kcnj16 Knockout Produces Audiogenic Seizures in the Dahl Salt-Sensitive Rat

Anna D. Manis[1,*], Oleg Palygin[1,2,*], Elena Isaeva[1], Vladislav Levchenko[1], Peter S. LaViolette[3], Tengis S. Pavlov[1,5], Matthew R. Hodges[1,2], Alexander Staruschenko[1,2,4,#]

[1]Department of Physiology, Medical College of Wisconsin, Milwaukee, WI 53226, USA

[2]Cardiovascular Center, Medical College of Wisconsin, Milwaukee, WI 53226, USA

[3]Radiology, Medical College of Wisconsin, Milwaukee, WI 53226, USA

[4]Clement J. Zablocki VA Medical Center, Milwaukee, WI 53295, USA

[5]Current address: Division of Hypertension and Vascular Research, Henry Ford Health System, Detroit, MI 48202, USA

*equal contribution

[#]To whom correspondence should be addressed: Alexander Staruschenko, PhD; Department of Physiology, Medical College of Wisconsin, 8701 Watertown Plank Road, Milwaukee, WI 53226, USA. Phone: (414) 955-8475; Fax: (414) 955-6546; E-mail: staruschenko@mcw.edu

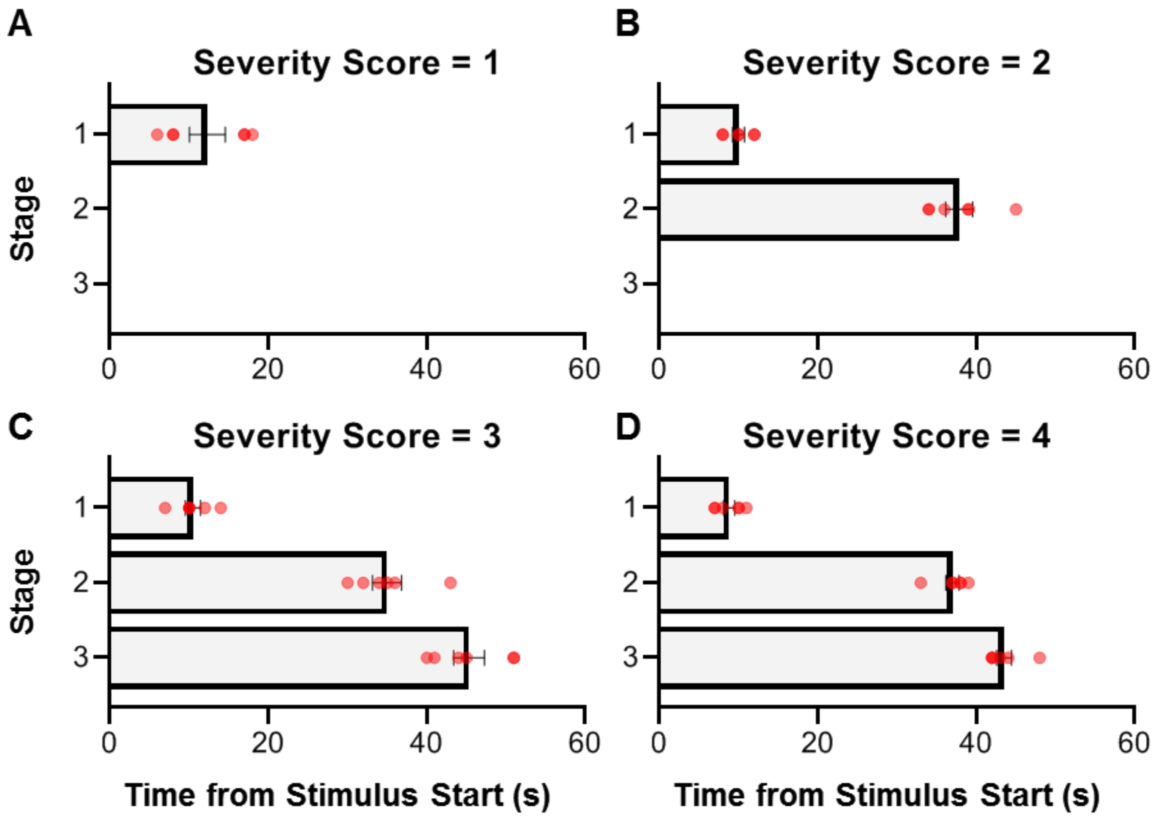

Figure S1. Latency of progressive behavioral stages for each seizure score (1-4). The time from the start of stimulus (in seconds) to each behavioral stage (described in Fig. 1A) is shown for seizures scoring 1-4 (shown in A-D, respectively). Data for individual rats (N=6 males) are indicated by the red dots.

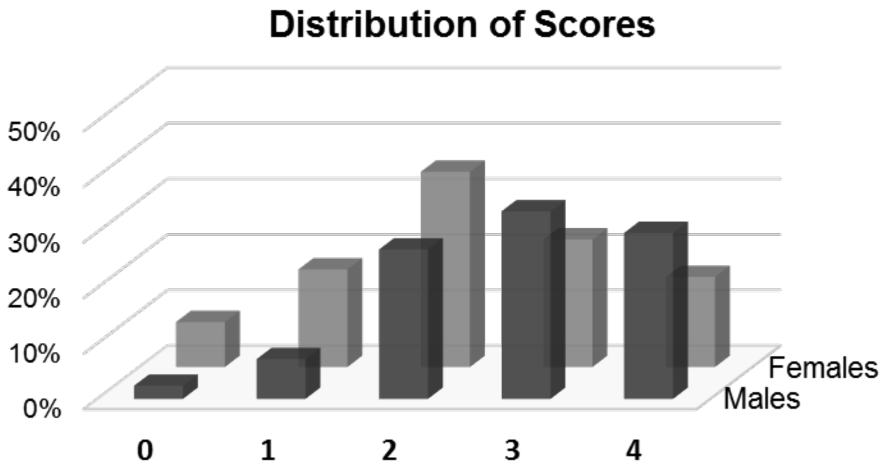

Figure S2. Distribution of seizure severity scores in male versus female $SS^{Kcnj16-/-}$ rats. The chart depicts the relative frequency distributions of observations of each seizure severity score in males (black bars, N=21) compared to females (gray bars, N=7).

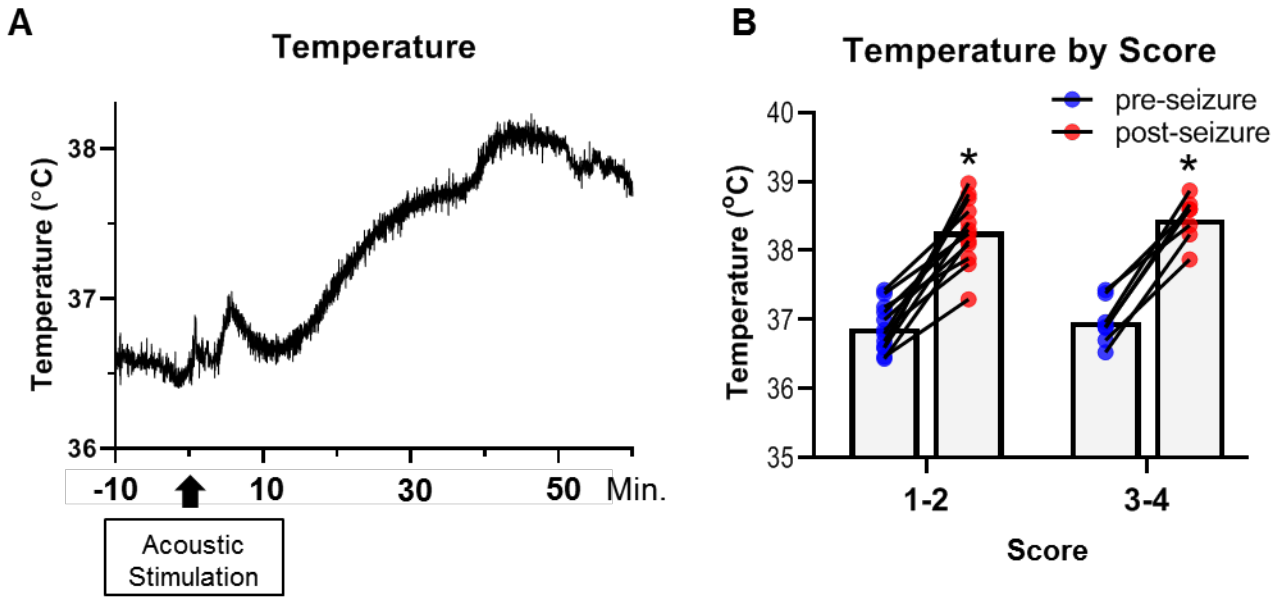

Figure S3. Postictal temperature elevations SS$^{Kcnj16-/-}$ rats. Core temperature was continually monitored using radiotelemeters implanted in the abdominal cavity before, during, and after seizure induction (N=4). (**A**) A representative trace depicting temperature increases over time in the postictal period. The acoustic stimulation occurs at time=0 minutes. (**B**) A summary of temperature changes (N=20 seizures represented) across seizure severity scores with paired pre (blue) and post (red) measurements indicated by a connecting line. Asterisks indicate significant ($p<0.001$) increase in temperature. The magnitude of temperature increase was not dependent on seizure severity score ($p=0.335$).

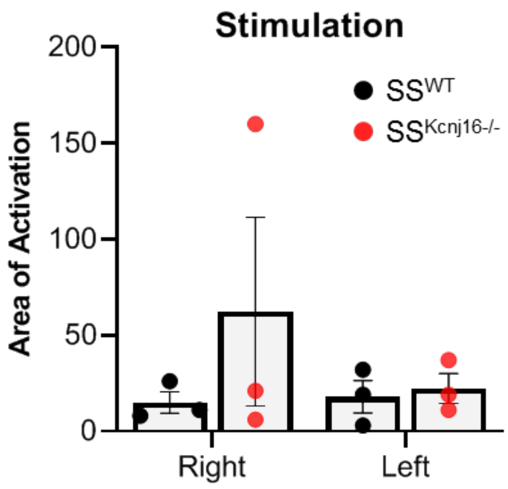

Figure S4. fMRI analysis of forepaw stimulation. No differences between SS^{WT} and $SS^{Kcnj16-/-}$ rats (N=3,4) were observed for electrical stimulation of either the right or left forepaw. The area of activation refers to activated voxels above control with 0.01 significance threshold. Error bars are SEM.

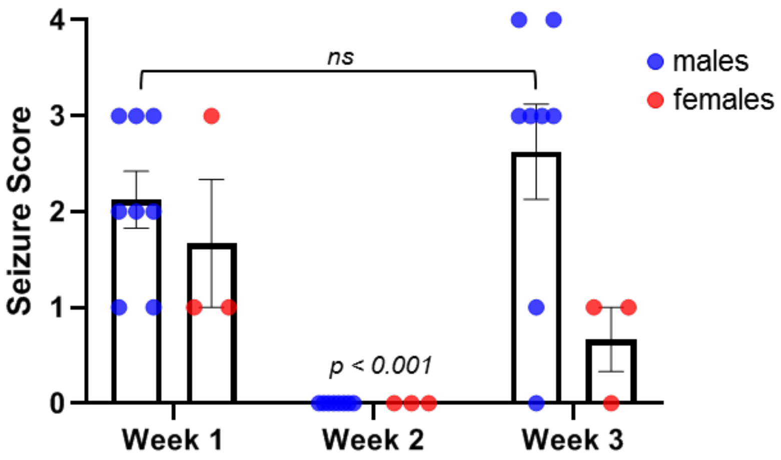

Figure S5. Pretreatment with diazepam prevents audiogenic seizures. Male (N=8, blue) and female (N=3, red) $SS^{Kcnj16-/-}$ rats were exposed to the acoustic stimulus once per week for 3 weeks. Rats were pretreated with 2.5 mg/kg, i.p. diazepam approximately 1 hr. prior to stimulation during week 2 and received vehicle injections before stimulations during weeks 1 and 3. Data points represent individual scores, bars represent the group mean severity per day, and error bars are SEM. Diazepam pretreatment prevented seizures during week 2 ($p < 0.001$).

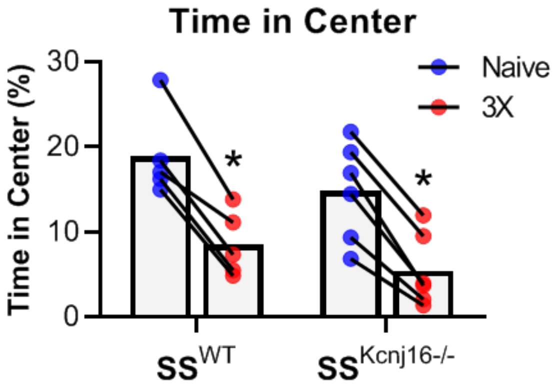

Figure S6. Open field test before and after 3 exposures to the acoustic stimulus in SS^{WT} and SS$^{Kcnj16-/-}$ rats. The chart portrays the percentage of time the rat spent in the center of the open field (as opposed to time spent along the perimeter which indicates anxiety behavior). Connecting lines indicate paired values comparing open field test results before any seizure exposure (Naïve; blue) and 3 exposures (3X, red). Changes observed were not due to seizure exposure, as both SS^{WT} and SS$^{Kcnj16-/-}$ rats showed similar decreases after 3X exposure to the acoustic stimulus.

		SS^{WT} Rats										SS$^{Kcnj16-/-}$ Rats											
		Naïve					3X Exposed					Naïve						3X Exposed					
Physical Factors and Gross Appearance	Coat color	A	A	A	A	A	A	A	A	A	A	A	A	A	A	A	A	A	A	A	A	A	A
	Sex	M	M	M	M	M	M	M	M	M	M	M	M	M	M	M	M	M	M	M	M	M	M
	Presence of whiskers	0	0	0	0	0	0	0	0	0	0	0	0	0	0	0	0	0	0	0	0	0	0
	Appearance of fur	0	0	0	0	0	0	0	0	0	0	0	0	0	0	0	0	0	0	0	0	0	0
	Piloerection	0	0	0	0	0	0	0	0	0	0	0	0	0	0	0	0	0	0	0	0	0	0
	Fur missing on face	0	0	0	0	0	0	0	0	0	0	0	0	0	0	0	0	0	0	0	0	0	0
	Fur missing on body	0	0	0	0	0	0	0	0	0	0	0	0	0	0	0	0	0	0	0	0	0	0
	Wounds	0	0	0	0	0	0	0	0	0	0	0	0	0	0	0	0	1	0	1	0	0	1
	Skin color	0	0	0	0	0	0	0	0	0	0	0	0	0	0	0	0	0	0	0	0	0	0
	Palpebral closure	0	0	0	0	0	0	0	0	0	0	0	0	0	0	0	0	0	0	0	0	0	0
Behavior in Novel Environment	Transfer behavior	0	0	0	0	0	0	0	0	0	0	0	0	0	0	0	0	1	1	0	0	0	0
	Body position	0	0	0	0	0	0	0	0	0	0	0	0	0	0	0	0	0	0	0	0	1	0
	Spontaneous activity	0	0	0	0	0	0	0	0	0	0	0	0	0	0	0	0	3	0	0	0	0	0
	Respiration rate	0	0	0	0	0	0	0	0	0	0	0	0	0	0	0	0	1	0	0	1	1	0
	Tremor	0	0	0	0	0	0	0	0	0	0	0	0	0	0	0	0	1	1	0	1	2	0
	Piloerection	0	0	0	0	0	0	0	0	0	0	0	0	0	0	0	0	0	0	0	0	0	1
	Gait	0	0	0	0	0	0	0	0	0	0	0	0	0	0	0	0	0	0	1	0	0	1
	Pelvic elevation	0	0	0	0	0	0	0	0	0	0	0	0	0	0	0	0	0	0	0	0	0	0
	Tail elevation	0	0	0	0	0	0	0	0	0	0	0	0	0	0	0	0	0	0	0	0	0	0
Reactions to Stimuli	Touch escape	0	0	0	0	0	0	0	0	0	0	0	0	0	1	0	0	0	1	0	0	1	0
	Positional passivity	0	1	0	0	0	0	0	0	0	0	0	0	0	0	0	0	0	0	0	0	0	0
	Reaching reflex	0	0	0	0	0	0	0	0	0	0	0	0	0	0	0	0	0	0	0	0	0	0
	Preyer reflex	0	0	0	0	0	0	0	0	0	0	0	0	0	0	0	0	0	0	0	0	0	0
	Air righting reflex	0	0	0	0	0	0	0	0	0	0	0	0	0	0	0	0	0	0	0	0	0	0
During Handling	Limb tone	0	0	0	0	0	0	0	0	0	0	0	0	0	0	0	0	0	0	0	0	0	0
	Abdominal tone	0	0	0	0	0	0	0	0	0	0	0	0	0	0	0	0	0	0	0	0	0	0
	Body tone	0	0	0	0	0	0	0	0	0	0	0	0	0	0	0	0	0	0	0	0	0	0
	Handling Score	0	0	0	0	2	0	1	0	0	1	1	2	1	1	1	1	2	3	1	1	2	2
	Rat Demeanor	0	0	0	0	0	0	0	0	0	1	2	1	2	1	1	0	2	3	1	0	0	2

Figure S7. Table summarizing Irwin screen results for SS^{WT} and SS$^{Kcnj16-/-}$ rats before and after exposure to 3X acoustic stimulations. Scores of 0 generally represent a "normal" result for each parameter. The scoring rubric used is included in the supplement. N=5 SS^{WT} and N=6 SS$^{Kcnj16-/-}$ rats were assessed.

		SS$^{Kcnj16-/-}$ Rats																					
		Naïve											10X Exposed										
Physical Factors and Gross Appearance	Coat color	A	A	A	A	A	A	A	A	A	A	A	A	A	A	A	A	A	A	A	A	A	A
	Sex	M	M	M	M	M	M	M	F	F	F	F	M	M	M	M	M	M	M	F	F	F	F
	Presence of whiskers	0	0	0	0	0	0	0	0	0	0	0	0	0	0	0	0	0	0	0	0	0	0
	Appearance of fur	0	0	0	0	0	0	0	0	0	0	0	1	0	1	0	0	1	2	0	0	0	0
	Piloerection	0	0	0	0	0	0	0	0	0	1	0	1	2	1	0	1	0	1	0	0	0	0
	Fur missing on face	0	0	0	0	0	0	0	0	0	0	0	0	0	0	0	0	0	0	0	0	0	0
	Fur missing on body	0	0	0	0	0	0	0	0	0	0	0	0	0	0	0	0	0	0	0	0	0	0
	Wounds	0	0	0	0	0	0	0	0	0	0	0	1	2	1	0	1	1	1	0	0	0	0
	Skin color	0	0	0	0	0	0	0	0	0	0	0	0	0	1	0	1	0	2	0	0	0	0
	Palprebal closure	0	0	0	0	0	0	0	0	0	0	0	0	0	0	0	0	0	1	0	0	0	0
Behavior in Novel Environment	Transfer behavior	0	0	0	0	0	0	0	0	0	0	1	0	0	0	0	0	0	2	0	0	1	1
	Body position	0	0	0	0	0	0	0	0	0	0	0	0	0	1	0	0	0	1	0	0	0	0
	Spontaneous activity	0	0	0	0	0	0	0	0	0	0	0	0	0	0	0	0	0	3	0	0	1	1
	Respiration rate	0	0	0	0	0	0	0	0	0	0	0	0	0	0	0	0	0	2	0	0	0	0
	Tremor	0	0	0	0	0	0	0	0	0	0	0	1	0	0	0	0	1	0	0	0	0	1
	Piloerection	0	0	0	0	0	0	0	0	0	0	0	0	0	1	0	0	0	2	0	0	0	0
	Gait	0	0	0	0	0	0	0	0	0	0	0	0	*	0	0	0	0	0	0	0	0	0
	Pelvic elevation	0	0	0	0	0	0	0	0	0	0	0	0	0	0	0	0	0	0	0	0	0	0
	Tail elevation	0	0	0	0	0	0	0	0	0	0	0	0	0	0	0	0	0	0	0	0	0	0
Reactions to Stimuli	Touch escape	0	0	0	0	0	0	0	0	0	0	0	0	0	0	0	0	0	1	0	0	0	0
	Positional passivity	0	0	0	0	0	0	0	0	0	0	0	0	0	0	0	0	0	0	0	0	0	0
	Reaching reflex	0	0	0	0	0	0	0	0	0	0	0	0	0	0	0	0	0	0	0	0	0	0
	Preyer reflex	0	0	0	0	0	0	0	0	0	0	0	0	0	0	0	0	0	0	0	0	0	0
	Air righting reflex	0	0	0	0	0	0	0	0	0	0	0	0	0	0	0	0	0	0	0	0	0	0
During Handling	Limb tone	0	0	0	0	0	0	0	0	0	0	0	0	0	0	0	0	0	0	0	0	0	0
	Abdominal tone	0	0	0	0	0	0	0	0	0	0	0	0	0	0	0	0	0	0	0	0	0	0
	Body tone	0	0	0	0	0	0	0	0	0	0	0	0	0	0	0	0	0	0	0	0	0	0
	Handling Score	0	0	1	1	1	1	1	1	1	2	0	1	1	2	0	0	0	0	1	0	0	1
	Rat Demeanor	1	1	1	1	1	1	1	1	2	1	1	1	2	1	1	1	1	2	3	1	3	3

Figure S8. Summary of Irwin screen results for SS$^{Kcnj16-/-}$ rats before and after exposure to 10X acoustic stimulations. Scores of 0 generally represent a "normal" result for each parameter. Scoring rubric is included in the supplement. N=7 male and 4 female SS$^{Kcnj16-/-}$ rats were assessed.

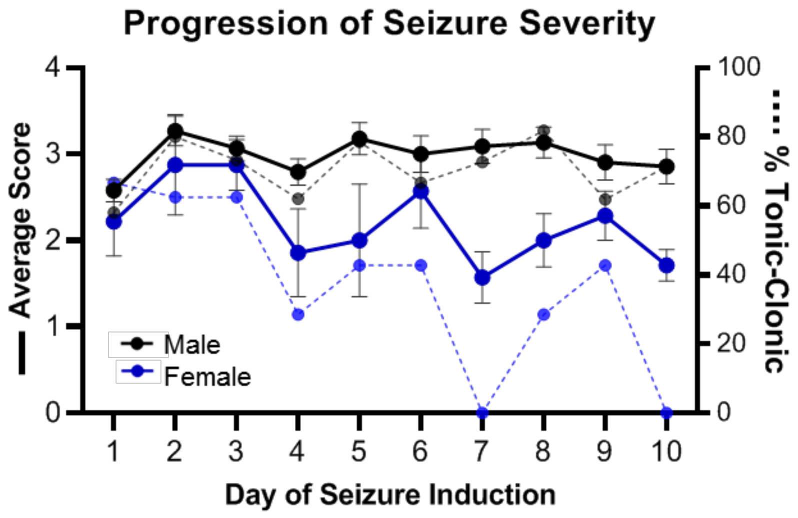

Figure S9. Progression of seizure severity with 10 days of repeated seizure induction in male and female $SS^{Kcnj16-/-}$ rats. The left y-axis (graphed as a solid line) indicates the average seizure severity on each of the 10 days of seizure induction for male (black; N=31) and female (blue; N=9) rats. The right y-axis (graphed as a broken line) denotes the percentage of seizures per day that reached tonic-clonic scores of 3 or 4. Seizure severity was not dependent on the number of seizure exposures (1-10).

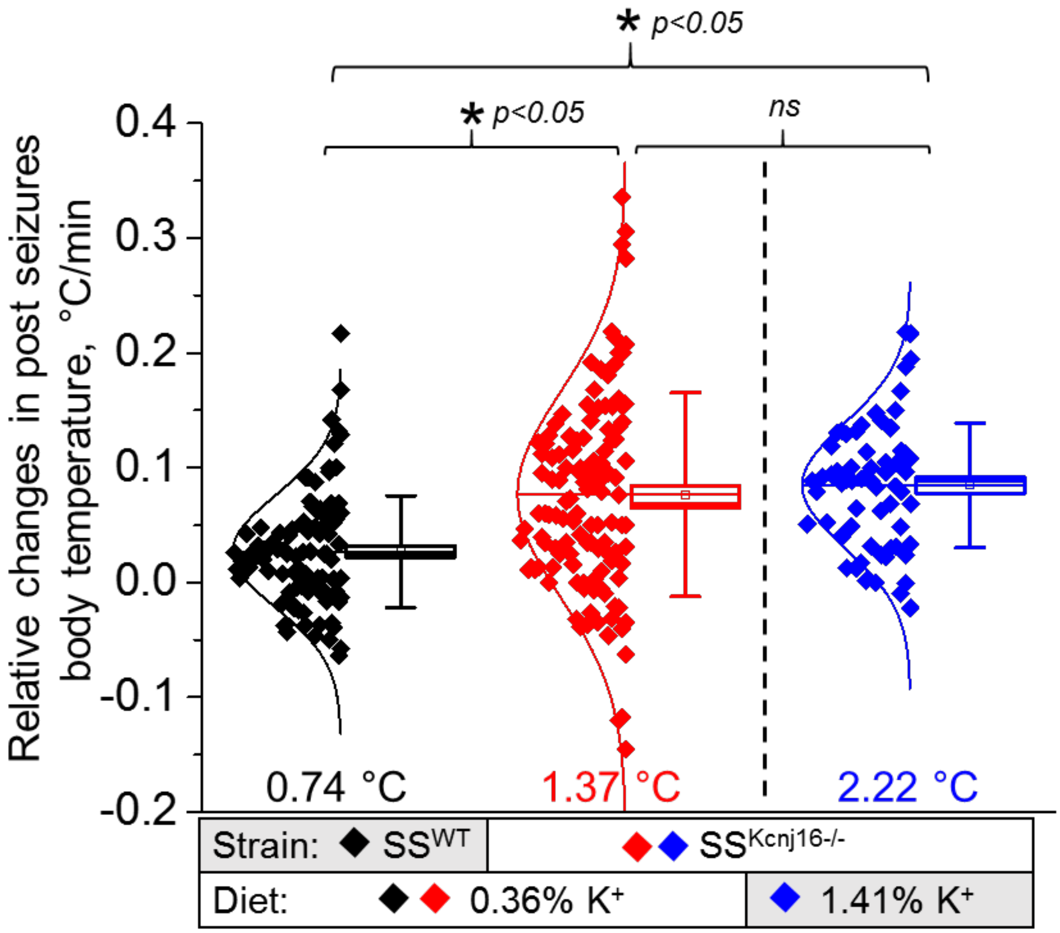

Figure S10. Postictal body temperature increases were unaffected by HKD. Rectal temperature was measured before and approximately 30 min after acoustic stimulation in SS^{WT} (black) and $SS^{Kcnj16-/-}$ rats fed a NKD (red) or HKD (blue). Temperature changes are shown relative to time from the acoustic stimulus. Relative changes in temperature were significantly greater in $SS^{Kcnj16-/-}$ rats fed either diet ($p<0.05$) compared to SS^{WT}.

Table S1. Supplemental methods- Irwin screen rubric

1	Physical factors and gross appearance	score	description
A	Coat Color	A	Albino
		Ag	Agouti
		Bl	Black
B	Presence of whiskers	0	Normal; full set
		1	Some missing
		2	All or nearly all missing
C	Appearance of fur	0	Well-groomed (normal)
		1	Somewhat disheveled
		2	Ungroomed and disheveled
D	Piloerection	0	none
		1	Most hairs standing on end
		2	Extreme piloerection
E	Patches of missing fur on face	0	none
		1	some
		2	extensive
F	Patches of missing fur on body	0	none
		1	some
		2	extensive
G	Wounds	0	none
		1	some
		2	extensive
H	Skin color	0	Pink (normal)
		1	white
		2	cyan
I	Palpebral Closure	0	Eyes wide open
		1	Eyes 1/2 closed
		2	Eyes closed
		3	Cataracts (note if one or both eyes)
**2**	**Observations in a novel environment**	**score**	**description**
A	Transfer behavior	0	Normal; brief freeze, then normal movement
		1	Extended freeze or moderately “manic”
		2	Immobile/extremely “manic”

B	Body position	0	Normal; sitting or standing
		1	Lying down, hunching, or excessive rearing
		2	Flat on stomach or repeated jumping
C	Spontaneous activity	0	Normal
		1	Vigorous scratch, groom, moderate movement
		2	Extremely vigorous, rapid/dart movement
		3	completely immobile
D	Respiration rate	0	Normal
		1	Moderately slow/fast
		2	Extremely slow/fast
E	Tremor	0	Mild
		1	None
		2	Marked
F	Piloerection	0	None
		1	Mild
		2	Excessive
G	Gait	0	Normal
		1	Abnormal or limited
		2	Incapacity
H	Pelvic Elevation	0	Normal
		1	Markedly flattened
		2	Barely Touches
		3	Elevated more than normal
G	Tail Elevation	0	Horizontally Extended (normal)
		1	Dragging
		2	Elevated (Straub tail)
**3**	**Reflexes and reactions to simple stimuli**	**score**	**description**
A	Touch Escape	0	Normal Escape
		1	No escape or extreme response to touch
B	Positional passivity	0	Struggles when restrained by tail (normal)
		1	Does not struggle
C	Reaching reflex	0	Normal; before vibrissae contact
		1	Upon vibrissae or nose contact
		2	none

D	Pinna reflex	0	Normal; ear retracts or flicks
		1	No response
E	Preyer reflex	0	Normal; ear flicks, rat flinches or jumps
		1	No response
F	Inverted Screen (s)	#	
G	Air Righting Reflex	0	No impairment
		1	No righting reflex
**4**	**Measures recorded during handling**	**score**	**description**
A	Limb Tone	0	Normal
		1	None or rigid (note which)
B	Abdominal Tone	0	Normal
		1	Slightly flaccid or stiff (note which)
		2	Extremely flaccid or stiff (note which)
C	Body tone	0	Normal; slight resistance
		1	Mildly flaccid or resistant (note which)
		2	Extremely resistant or flaccid (note which)
D	Ease of Handling	0	Easy to handle/ passive
		1	Normal struggling/escape
		2	very scared/agitated/aggressive
E	Demeanor	0	relaxed/passive
		1	curious/exploratory
		2	anxious/ overactive
		3	aggressive/ stressed
